# Evaluation of Sugarcane-Derived Polyphenols on the Pre-Weaning and Post-Weaning Growth of Gilt Progeny

**DOI:** 10.3390/ani10060984

**Published:** 2020-06-05

**Authors:** Udani A. Wijesiriwardana, John R. Pluske, Jessica R. Craig, Jeremy J. Cottrell, Frank R. Dunshea

**Affiliations:** 1 Faculty of Veterinary and Agricultural Sciences, The University of Melbourne, Parkville, VIC 3010, Australia; jcottrell@unimelb.edu.au (J.J.C.); fdunshea@unimelb.edu.au (F.R.D.); 2 Agricultural Sciences, College of Science, Health, Engineering and Education, Murdoch University, Murdoch, WA 6150, Australia; j.pluske@april.org.au; 3 Australasian Pork Research Institute Ltd. (APRIL), Willaston, SA 5118, Australia; 4 Research and Innovation, Rivalea (Australia) Pty. Ltd., Corowa, NSW 2646, Australia; jcraig@rivalea.com.au

**Keywords:** gilt progeny, sugarcane, polyphenols

## Abstract

**Simple Summary:**

Gilt progeny are characterised by their poor lifetime growth performance compared with sow progeny. Various feeding strategies that employ the use of additives may be used to improve their growth. Gilts are said to be in increased oxidative stress throughout lactation, which may contribute to the reduced growth performance seen in their progeny. Furthermore, weaning is associated with increased inflammation, which can reduce growth after weaning. In this study, both late gestation/lactation diets and weaner diets were supplemented with a sugarcane extract rich in polyphenols owing to their anti-oxidative and anti-inflammatory properties to collectively improve the growth of gilt progeny. However, no improvements of growth performance of gilt progeny in both the pre-weaning and post-weaning periods were observed in response to polyphenol supplementation and sow progeny continuously outperformed gilt progeny. Furthermore, when inflammation was measured using the inflammatory cytokine interleukin-1β, no differences were found between the control and polyphenol supplemented group. However, gilt progeny exhibited reduced circulating interleukin-1β overall. In summary, gilt progeny experience persistent underperformance that may be coupled in part to poorer immune development and polyphenol supplementation did not overcome the persistent underperformance.

**Abstract:**

Gilt progeny (GP) exhibit poorer growth compared with sow progeny (SP), particularly in the pre-weaning and post-weaning period. Late gestation/lactation sow diets and weaner diets were supplemented with 0.5% Polygain (POL), a sugarcane extract rich in polyphenols, to collectively improve GP growth in these periods. Gilts (*n* = 60) and sows (*n* = 68, parities 2 and 3) were fed a control or POL diet. Weaned GP (*n* = 79) and SP (*n* = 92) born to these dams were also fed either a CON or POL diet. Gilts litters weighed less than sow litters at birth and 21 days (*p* < 0.001 for both) and were not improved by POL (*p* = 0.80 and 0.54, respectively). GP were lighter than SP at day 7 and day 14 post-weaning (*p* < 0.001 for both) and were not improved by the POL diet at these timepoints (*p* = 0.61 and 0.97, respectively). Plasma interleukin-1β (IL-1β) was increased at weaning despite POL supplementation (*p* = 0.022) and GP had reduced IL-1β overall (*p* = 0.021). Overall, POL was unable to improve growth in GP and the attenuated immune response seen in GP could be contributing to their poor growth performance.

## 1. Introduction

Gilt progeny (GP) are characterised by low birth weights, slow growth, higher medication rates, and greater rates of post-natal mortality [1,2,3], resulting in significant losses for pork producers. Gilt replacement exceeds 50% of the Australian reproductive herd [4]; therefore, the success of their progeny is critical for pig production. Gilts are mated at a physiologically young age to reduce non-productive days, and so the gestating gilt must support her own continued growth and development throughout pregnancy. However, this often compromises the growth and development of her progeny and, as a result, GP consistently perform more poorly compared with multiparous sow progeny (SP) [3,5].

Gestation and lactation are energetically demanding periods for the dam that are associated with systemic oxidative stress [6]. This is more acute in the gilt than in the sow [7] and is linked to impaired milk production in the pre-weaning period [6]. The comparatively poorer growth of GP during the pre-weaning period [3] can partially be attributed to the lower milk yield of the gilt [8,9], meaning that reducing oxidative stress in lactating gilts may be a useful management strategy for increasing productivity in GP. This was demonstrated by Lipiński et al. [10], who observed antioxidant polyphenol supplementation improved litter and body weights at weaning. Polyphenols are secondary plant metabolites that exhibit anti-oxidative effects [11]. In addition to their antioxidative properties, polyphenols also exhibit anti-inflammatory properties [12].

Increased inflammation is often associated with weaning [13]. This is usually in response to the functional and structural changes of the gastrointestinal tract [14,15] caused by the abrupt shift of nutrients from highly digestible milk solids to complex cereal feed. Furthermore, this immediate post-weaning period is often characterised by reduced growth rates, which is particularly observed in GP [3,5]. Whether increased inflammation contributes specifically to the inferior post-weaning growth performance seen in GP is yet to be fully elucidated. However, supplementing weaner diets with plant polyphenols may reduce weaning associated inflammation [16,17] and potentially improve GP growth. Polygain (POL) is a sugarcane molasses-derived extract that is rich in polyphenols including chlorogenic acid, caffeic acid, sinapic acid, syringic acid, vanillin, homoorientin, orientin, vitexin, swertisin, diosmin, apigenin, tricin, and diosmetin, as well as a number of apigenin-, methoxyluteolin-, and tricin-O-glycosides [17]. Dietary POL has been shown to alleviate oxidative stress and increase growth rate of broiler chickens suffering heat stress [18].

This study aimed to evaluate the effects of POL supplementation on the growth of GP during the lactation and early post-weaning period. It was hypothesized that supplementing late gestation/lactation and weaner diets with POL would collectively improve pre-weaning and weaning performance in GP.

## 2. Materials and Methods

### 2.1. Ethics Statement

Experimental procedures were approved by the Rivalea (Australia) Pty Ltd. Animal Care and Ethics Committee (protocol number 18N006C) in accordance with the Australian Code for the Care and Use of Animals for Scientific Purposes (National Health and Medical Research Council, 2013).

### 2.2. Animals, Experimental Design, and Diets

The experiment was conducted under commercial conditions at a piggery in New South Wales, Australia (Rivalea Australia, Pty Ltd., Corowa, NSW, Australia), from March to April 2018.

#### 2.2.1. Gestation and Lactation Phases

The study consisted of a 2 × 2 factorial design (parity × diet) with a total of 60 gilts (parity 1) and 66 multiparous sows (parities 2 and 3; Large White × Landrace, Primegro^TM^ Genetics, Corowa, Australia) (2 ± 0.1 parities; mean ± SE) over two replicates. Gilts and sows were weighed and P2 backfat measurements were taken at day 110 upon entry to farrowing house and at weaning. Gilts and sows were then randomly allocated to a wheat- and canola meal-based diet (CON) (14.8 MJ digestible energy (DE)/kg, 16.5% crude protein (CP), 8.3% crude fat, 4.3% crude fibre, and 0.9% standardized ileal digestible (SID) Lys; as-fed basis) or the CON diet with 0.5% POL, with the POL replacing wheat. Diets were fed from day 110 (110 ± 0.2 days; mean ± SE) of gestation and throughout lactation (24.6 ± 0.1 days; mean ± SE) until weaning. Whole diets were analysed for total polyphenol content as per methods described by Deseo, et al. [19] and the addition of POL resulted in an 11% increase in total polyphenol content of the diet (2.71 vs. 2.44 mg/g). Gestating gilts and sows were fed 3.5 kg/day of their allotted diet at 09:00 h each day after entry to the farrowing house. Once farrowed, gilts and sows were fed 3.5 kg on the day of farrowing, 5 kg one day after farrowing, and ad libitum from the second day until weaning. Feed intake and refusals were recorded daily in this period. Gilts and sows were housed in the same farrowing room in alternating farrowing crates fitted with nipple drinkers for both dams and piglets, with piglets having access to a heat lamp in the creep area. There was no creep feed provided. All live piglets were individually weighed and tagged within 24 h of birth. The two median weight male focal piglets were selected from each litter for blood samples to be taken at 1 day prior to weaning. The number of born alive piglets, still born, and mummified foetuses were recorded for each litter before any cross fostering, which was carried out as per commercial procedures with piglets fostered within parity and diet where possible. At 21 days, piglets were individually weighed again. A total of 25 GP and 30 SP were used for inflammatory marker analysis for the pre-weaning period.

#### 2.2.2. Post-Weaning Phase

Piglets were weaned at approximately 24.6 (±0.1 days) days of age. The male focal piglets of each litter in lactation were relocated to individual pens in the weaner unit of the farm, while non-focal piglets continued on to regular commercial production. Weaner pigs were offered a common starter wheat, dehulled oats, and soybean meal-based diet (14.7 MJ DE/kg, 20.3% CP, 4.08% crude fat, 2.6% crude fibre, and 1.3% SID Lys; as-fed basis), with experimental diets containing 0.5% POL replacing wheat. The addition of POL resulted in an 8% increase in total polyphenol content of the diet (2.23 mg/g vs. 2.05 mg/g). Weaned pigs from dams that had been fed the control diet were also fed the control diet, whereas those that were from dams fed POL were also fed the POL diet. After weaning, weights were not taken upon entry to the weaner pens owing to resource constraints associated with the commercial batch weaning system. However, piglets were individually weighed at 7 and 14 days after weaning. Feed intake was recorded from weaning to 14 days, after which all focal piglets were returned to regular commercial production.

### 2.3. Inflammatory Marker Analysis

Blood samples were taken the day before and at 3 and 14 days after weaning. Pigs were mildly restrained in dorsal recumbency by a trained operator, and blood was then collected via jugular venepuncture using a 21-gauge needle for new-borns, and a 20-gauge needle for piglets aged 21 to 40 days, into lithium heparin vacutainer tubes (BD™ Vacutainer™, North Ryde, NSW, Australia). Each tube was inverted 3−4 times to ensure adequate mixing of the sample and anticoagulant. Tubes were then placed on ice until centrifugation for 10 min at 4 °C. Plasma was collected and stored at −20 °C until analysis. Piglet plasma was assayed for interleukin-1 beta (IL-1β) (DuoSet^®^ Porcine IL-1β/IL-1F2, catalogue number DY681, R&D systems, Minneapolis, MN, USA; 2–5% intra-assay covariation (CV) and 5% inter-assay CV), as per the manufacturer’s instructions.

### 2.4. Statistical Analysis

Liveweight, P2 backfat, litter weights, weaned piglet weights, feed intake, and circulating IL-1β were analysed by linear mixed models using GENSTAT (16th edition, Hemel Hempstead, UK). Dam parity (gilt vs. sow) and diet (control vs. POL) were the main and interactive factors for all analysis except circulating IL-1β. Circulating IL-1β was analysed using dam parity, diet, and time (pre-weaning, 3 days post-weaning, and 14 days post-weaning) as main and interactive factors. Replicate was designated as the random model. The sow was designated as the random model for post-weaning growth performance. Piglet age at weaning was designated as a covariate in the random model when analysing day 7 and day 14 post-weaning weights. Sow ID and Piglet ID were designated in the random model for IL-1β analysis. When interactive effects were found to be significant in IL-1β analysis, a post-hoc Fisher’s Least Significant Difference (LSD) test was performed for multiple pairwise comparisons. Chi-squared (*χ*^2^) tests were used to analyse pre-weaning mortality between parities and between diets. Data are presented as mean ± standard error of the differences (SED). A value of *p* < 0.05 was used to indicate statistical significance.

## 3. Results

### 3.1. Farrowing Performance

Gilts were lighter (202 vs. 276 kg, *p* < 0.001) and had lower P2 backfat (19.0 vs. 21.1 mm, *p* = 0.009) than sows at entry into the farrowing house at d110 of gestation (data not shown). Similarly, gilts were lighter (190 vs. 257 kg, *p* < 0.001) and had lower P2 backfat (19.0 vs. 22.0 mm, *p* < 0.001) than sows at weaning. There was no effect of dietary POL on either dam liveweight (*p* = 0.26) or P2 back fat (*p* = 0.55) at weaning. Dietary POL supplementation decreased lactation feed intake (6.61 vs. 7.15 kg/day, *p* = 0.001), while gilts had lower lactation feed intake than sows (7.18 vs. 7.84 kg/day, *p* = 0.002). Sows had more piglets born alive compared with gilts (12.2 vs. 11.2 pigs, *p* = 0.021) with no effect of diet (*p* = 0.61). There were no parity (*p* = 0.49) or dietary (*p* = 0.92) effects on the number of stillborn piglets. Similarly, there were no parity (*p* = 0.73) or dietary (*p* = 0.71) effects on the number of mummified piglets (Table 1).

### 3.2. Pre-Weaning Performance 

Gilts had a lighter total litter weight (13.7 vs. 18.3 kg, *p* < 0.001) and individual piglet weight (1.25 vs. 1.50 kg, *p* < 0.001) at birth compared with sows. Supplementation with POL had no effect on total litter weight (*p* = 0.80) at birth, but resulted in lower individual birth weight (1.34 vs. 1.41 kg, *p* = 0.029). There were no parity (*p* = 0.25) or dietary effects (*p =* 0.28) on intra-litter CV in birth weight. Gilts showed a lighter total litter weight (41.4 vs. 61.8 kg, *p* < 0.001) and individual piglet weight (4.68 vs. 6.31 kg, *p* < 0.001) at 21 days of age than multiparous sows. There were no effects of dietary POL on total litter weight (*p* = 0.25) or individual piglet weight (*p* = 0.30) at 21 days. Gilt litters had lower intra-litter weight CV at 21 days (19.7 vs. 23.2%, *p* = 0.006), but this was not affected by dietary POL (*p* = 0.68). Total weight gain was lower in litters from gilts (1.30 vs. 2.11 kg/day, *p* < 0.001), but was not altered by dietary POL (*p* = 0.48) (Table 1). There were no effects of dietary POL (11.2 vs. 11.6%, *χ*^2^ = 0.07, *p* = 0.79) or between GP and SP (11.8 vs. 11.0%, *χ*^2^ = 0.26, *p* = 0.61) on pre-weaning mortality.

### 3.3. Post-Weaning Performance

Gilt progeny were lighter than SP at day 7 (7.18 vs. 9.02 kg, *p* < 0.001) and day 14 after weaning (9.61 vs. 11.8 kg, *p* < 0.001). There was no effect of dietary POL on liveweight at day 7 (*p* = 0.61) or day 14 (*p* = 0.97) after weaning. Live weight gain was lower in GP compared with SP from day 7 to 14 (347 vs. 394 g/day, *p* = 0.042), but was unaffected by dietary POL (*p* = 0.40). Feed intake from weaning to day 7 was not affected by parity (*p* = 0.60) or diet (*p =* 0.77). Feed intake between days 7 and 14 after weaning was lower in GP that in SP (375 vs. 428 g/day, *p* = 0.018), regardless of diet (*p* = 0.67). There was no effect of either parity (*p* = 0.37) or dietary POL (*p* = 0.29) on feed conversion ratio between days 7 and 14 after weaning and no significant interactions were observed (*p* = 0.18) (Table 2). No mortalities were observed in the post weaning period.

### 3.4. Plasma IL-1β Concentrations

Plasma IL-1β concentrations were lower in GP than in SP (2264 vs. 2568 pg/mL, *p* = 0.021) and decreased after weaning (2556, 2449, and 2244 pg/mL at pre-weaning, 3 days post-weaning, and 14 days post-weaning, respectively, *p* = 0.002), while no main effect of dietary POL (2354 vs. 2478 pg/mL for CON and POL, respectively, *p* = 0.15) was observed. However, there were significant interactions between diet and parity (*p* = 0.041), such that CON GP had lower plasma IL-1β concentrations than GP from dams supplemented with POL (Table 3), while no significant differences were observed between CON SP and POL SP. Moreover, the effect of parity was most pronounced at 14 days after weaning, where plasma IL-1β concentrations were 35% higher in SP than in GP (2577 vs. 1910 pg/mL), although there were no differences in pre-weaning and 3 days post-weaning (Table 3).

## 4. Discussion

This study confirmed that GP perform more poorly than SP during the pre-weaning and immediate post-weaning period. Moreover, feeding POL to primiparous sows in gestation and lactation did not improve pre-weaning and post-weaning performance of their progeny. Furthermore, the POL diet was unable to reduce plasma IL-1β at weaning and GP had lower plasma IL-1β at 14 days post-weaning compared with SP. An additional finding was that GP had reduced concentrations of the inflammatory marker IL-1β overall, possibly indicating delayed development of the immune system.

During the pre-weaning period, litters born to gilts were lighter than those born to sows, and this extended to 21 days and beyond. Furthermore, gilt litters grew slower compared with sow litters, which exacerbated the weight difference later on, which is consistent with the literature [20]. Low birth weights can limit pre-weaning and post-weaning growth [21,22], which was seen in this study. Furthermore, while gilts and sows have similar milk composition [9], their progeny grow at different rates and, therefore, this poor pre-weaning growth must be partially attributed to lower milk yield of the gilt [8]. Individual piglet and litter weight are strong drivers of dam milk production [23], hence the reduction in suckling pressure from the lighter GP may further contribute to the lower milk production of gilts.

Gilt progeny were lighter at day 7 after weaning and grew slower from day 7 to day 14 after weaning in comparison with SP. Slower growth after weaning has been previously reported especially in low birth- and weaning-weight piglets [24,25], and is often attributed to the abrupt shift from highly digestible milk constituents to more complex solid feed. This post-weaning growth check seems to be exacerbated in GP, reflected particularly by the reduced feed intake and live weight gain in this study, which has been previously reported [20]. Interestingly, there was no difference in feed intake between GP and SP in the immediate 7-day post-weaning period. However, there was a clear difference in the second week following weaning, where SP consumed 14% more feed than GP. This further validates the impacts of the post-weaning growth check that newly weaned pigs experience, but also demonstrates the ability of SP to overcome the challenge of weaning more quickly than GP. In this context Pluske, et al. [26] found that light pigs at weaning have a less developed gastrointestinal tract than heavier pigs and that there was a progressive increase in small intestinal brush border maltase, glucoamylase and sucrose, and pancreatic trypsin activity over the first 2 weeks after weaning, whereas brush border lactase activity declined in these light piglets. These changes in enzyme activities reflect the transition from a liquid milk-based diet to a complex solid cereal-based diet and indicate that heavier pigs are better able to make this adjustment, which may have been the case of SP in comparison with GP.

A key finding of this study was that GP had increased concentrations of IL-1β at 3 days post-weaning, but reduced concentrations overall compared with SP, regardless of POL supplementation. This was especially evident at day 14 following weaning and was largely owing to the maintenance of plasma IL-1β concentrations across the entire period in SP, whereas there was a decrease with time in GP. While SP had increased IL-1β concentrations at this timepoint, they also ate more and grew faster than GP. On the basis of the concentrations of circulating IL-1β found in this study along with their consistent underperformance, it seems that GP display compromised immune development. While this must be viewed as a preliminary observation, it seems that slow growing piglets displayed impaired transcription of genes associated with acquired and innate immunity [27]. Furthermore, studies of foetuses that suffer from intrauterine growth restriction (IUGR) have lower circulating cytokines in comparison with non-IUGR foetuses [28], with IUGR piglets having reduced circulating IL-1β concentrations in comparison with non IUGR piglets at 21 days of age [29]. Gilt progeny have been reported to exhibit a degree of IUGR owing to their asymmetric organ and tissue growth patterns [30], and this may contribute to the attenuated immune responses seen in GP. Of course, there are other pro-inflammatory markers that could be been investigated such as Tumour Necrosis Factor Alpha (TNF-α), which has been shown to increase markedly in response to lipopolysaccharide (LPS) challenge in weaned pigs [31], and these should be investigated in future studies.

Gilts and sows supplemented with the POL diet ate less than their control counterparts, and this may be in part owing to its bitter flavour profile [32], a result of the high concentration of phenolic compounds [32]. However, the POL diet did not reduce feed intake after weaning as it did for gilts and sows. The lack of dietary effect on feed intake in weaned piglets compared with their dams may have been owing to the exposure of the POL diet before weaning [33,34,35]. It is possible that the amount of supplemental POL was insufficient to increase dietary polyphenols adequately to elicit any production against oxidative stress. Alternatively, the background polyphenol concentrations in the control diets may have been too high to mask any effects of POL. The dam and weaner diets contained canola meal and/or soybean meal, both of which are rich sources of polyphenols [36,37]. In this context, dietary POL increased growth performance and improved meat quality in broiler chickens, with responses being most pronounced in the finisher period and when chickens were in a state of oxidative stress induced by high temperatures [18]. The responses were dose-dependent in a linear manner up to a dose that was twice as high as that used in the present study. Encouragingly, a holly extract rich in polyphenols and tannins has been shown to alleviate gastrointestinal inflammation in LPS challenged weaned piglets with reduced IL-6 and TNF-α concentrations being reported [38]. Furthermore, holly supplemented diets altered intestinal microbiota in LPS challenged weaned piglets and improved their intestinal barrier function with upregulation of tight junction proteins such as claudin-1 and occludin observed [38]. These results were seen in response to an inclusion of a commercial holly polyphenol product containing 65.5% total polyphenols. Therefore, it is proposed that further studies should investigate higher inclusion rates and should focus on weaner pigs.

## 5. Conclusions

Dietary supplementation of POL did not improve GP growth performance with GP performing poorly in the pre-weaning and post-weaning period compared with SP. The POL diet was unable to reduce IL-1β in GP and SP overall. By the end of the weaner component of this study, GP weighed less, ate less, and had reduced plasma IL-1β. Collectively, this indicates that GP may exhibit reduced immunocompetence compared with SP, and further work is required in characterizing the immune response of GP.

## Figures and Tables

**Table 1:** The effect of parity (gilt vs. sow) and diet (control, CON vs. 0.5% dietary Polygain, POL) on gestation and lactation performance of dams and their progeny. SED, standard error of the differences.

Parity (P)	GILT		SOW		SED	*p*-Value		
Diet (D)	CON	POL	CON	POL		P	D	P × D
*(n)*	29	31	34	33				
Dam weaning liveweight (kg)	189	190	259	255	6.4	<0.001	0.26	0.58
Dam weaning P2 backfat (mm)	20.0	18.0	21.5	22.4	1.33	0.001	0.55	0.14
Dam lactation feed intake (kg/day)	6.93	6.19	7.38	7.04	0.236	<0.001	0.002	0.24
Piglets born alive (#/L)	10.9	11.3	12.1	12.3	0.66	0.020	0.61	0.82
Piglets still born (#/L)	0.92	1.06	1.23	1.17	0.429	0.49	0.92	0.74
Mummified piglets (#/L)	0.18	0.30	0.31	0.12	0.128	0.73	0.71	0.09
Birth litter weight (kg)	13.8	13.6	18.4	18.3	0.86	<0.001	0.80	0.89
Birth piglet weight (kg)	1.29	1.20	1.55	1.49	0.049	<0.001	0.029	0.64
Birth weight intra-litter CV (%)	17.8	18.1	18.3	20.3	1.63	0.25	0.28	0.48
21-day litter weight (kg)	42.2	40.7	59.7	64.0	3.59	<0.001	0.54	0.25
21-day piglet weight (kg)	4.85	4.60	6.37	6.26	0.239	<0.001	0.30	0.68
21-day weight intra-litter CV (%)	18.6	20.8	23.7	22.7	1.72	0.006	0.68	0.19
Litter daily gain (kg/day)	1.33	1.27	2.04	2.17	0.113	<0.001	0.48	0.18

**Table 2:** The effect of parity (gilt vs. sow) and diet (control, CON vs. 0.5% dietary Polygain, POL) on the post-weaning performance of dam progeny.

Parity (P)	GILT		SOW		SED	*p*-Value		
Diet (D)	CON	POL	CON	POL		P	D	P × D
*(n)*	47	32	49	42				
Day 7 liveweight (kg)	7.19	7.17	9.17	8.86	0.359	<0.001	0.61	0.58
Day 14 liveweight (kg)	9.66	9.55	11.8	11.8	0.49	<0.001	0.97	0.88
Day 7−14 live weight gain (g/day)	353	341	372	415	30.9	0.042	0.40	0.20
Day 0−7 feed intake (g/day)	184	181	185	193	16.1	0.60	0.77	0.59
Day 7−14 feed intake (g/day)	385	366	414	443	29.8	0.018	0.67	0.25
Day 7−14 feed conversion ratio	1.12	1.16	1.32	1.11	0.129	0.37	0.29	0.18

**Table 3:** The effect of parity (gilt progeny (GP) vs. sow progeny (SP)), diet (control, CON vs. 0.5% dietary Polygain, POL) and time (pre-weaning, PreW vs. 3 days post weaning, 3 days PoW vs. 14 days post-weaning, 14 days PoW) on interleukin-1β (IL-1β) concentrations in plasma of progeny.

Diet (D)	Parity (P)	Timepoint (T)			SED	Significance ^1^
		PreW	3 Days PoW	14 Days PoW		
CON	GP	2190	1982	1838	320	P ^+^, T **, D × P *, D × T *, P × T *
	SP	2830	2555	2728		
POL	GP	2584	3010	1982		
	SP	2618	2250	2427		

^1^*p ^+^* < 0.10; * *p* < 0.05; ** *p* < 0.01; Other main and interactive effects *p* > 0.10.
